# Domain Expansion and Functional Diversification in Vertebrate Reproductive Proteins

**DOI:** 10.1093/molbev/msac105

**Published:** 2022-05-19

**Authors:** Alberto M. Rivera, Damien B. Wilburn, Willie J. Swanson

**Affiliations:** Department of Genome Sciences, University of Washington, Seattle, WA, USA; Department of Genome Sciences, University of Washington, Seattle, WA, USA; Department of Biomedical Informatics, The Ohio State University, Columbus, OH, USA; Department of Genome Sciences, University of Washington, Seattle, WA, USA

**Keywords:** fertilization, gene duplication, molecular evolution, machine learning, phylogenetics, protein structure

## Abstract

The rapid evolution of fertilization proteins has generated remarkable diversity in molecular structure and function. Glycoproteins of vertebrate egg coats contain multiple zona pellucida (ZP)-N domains (1–6 copies) that facilitate multiple reproductive functions, including species-specific sperm recognition. In this report, we integrate phylogenetics and machine learning to investigate how ZP-N domains diversify in structure and function. The most C-terminal ZP-N domain of each paralog is associated with another domain type (ZP-C), which together form a “ZP module.” All modular ZP-N domains are phylogenetically distinct from nonmodular or free ZP-N domains. Machine learning–based classification identifies eight residues that form a stabilizing network in modular ZP-N domains that is absent in free domains. Positive selection is identified in some free ZP-N domains. Our findings support that strong purifying selection has conserved an essential structural core in modular ZP-N domains, with the relaxation of this structural constraint allowing free N-terminal domains to functionally diversify.

## Introduction

Protein structural domains are a major type of molecular building block that multimerize into higher-order assemblies and provide the architectural foundation for nearly all cellular features, including organelles and extracellular matrices. Within molecular complexes, structural domains function as interlocking modules with specific, well-defined binding surfaces. Consequently, structural proteins commonly experience intense purifying selection to preserve their 3D conformations, which can lead to extreme sequence conservation between diverse taxa (e.g., actin is 89% identical between yeast and humans) (Rivero and Cvrčková 2007). The modularity of structural domains makes them prime templates for duplication within a genome, taking the form of both whole gene duplication to produce new paralogs and the formation of tandem domain arrays within a single gene (Rivera and Swanson 2022). Redundancy of duplicated domains can relax purifying selection to allow for diversification and neofunctionalization, as is observed for the mechanosensitive tandem domains of cadherins in the inner ear (Jaiganesh et al. 2018). However, little is known as to how positive selection can shape structural domain diversification within rapidly evolving systems.

Within animal genomes, many of the fastest evolving genes are associated with fertilization (Swanson and Vacquier 2002). Although often considered paradoxical, reproductive proteins evolve at extraordinary rates in part due to differences in male and female optimal mating rates that can drive sexual arms races, especially in gamete recognition proteins that initially mediate sperm–egg interactions (Wilburn and Swanson 2016; Wilburn et al. 2019). Fertilization of an egg by multiple sperm will fail to form a zygote—a phenomenon known as pathological polyspermy—and oocytes possess multiple reproductive barriers to modulate the rate of sperm entry (Frank 2000; Carlisle and Swanson 2021). One such barrier in vertebrate oocytes is an elevated glycoprotein envelope with clade-specific names: the zona pellucida (ZP) in mammals, the chorion in fishes, and the vitelline membrane in amphibians, reptiles, and birds (Wilburn and Swanson 2018). Named after the mammalian version, all vertebrate egg coat proteins contain a pair of immunoglobulin-like domains, ZP-N and ZP-C, that together form a polymerization unit called a ZP module (Jovine et al. 2002; Wilburn and Swanson 2017; Bokhove and Jovine 2018). The last common ancestor of vertebrates possessed six paralogous genes (*zp1*, *zp2*, *zp3*, *zp4*, *zpd*, and *zpax*) that experienced clade-specific birth and death events. Consequently, the egg coat of each major vertebrate class has a different composition of ZP module–containing proteins (Conner et al. 2005; Wong and Wessel 2005; Goudet et al. 2008; Meslin et al. 2012; Shu et al. 2015; Wassarman and Litscher 2016; Killingbeck and Swanson 2018). ZP modules are also found in nonreproductive proteins that form extracellular matrices, such as uromodulin (UMOD), which protects against urinary pathogens (Brunati et al. 2015; Bokhove et al. 2016; Devuyst and Pattaro 2018) and tectorin alpha (TECTA), which function in inner ear organization (Bokhove et al. 2016; Kim et al. 2019).

**Fig. 1.**
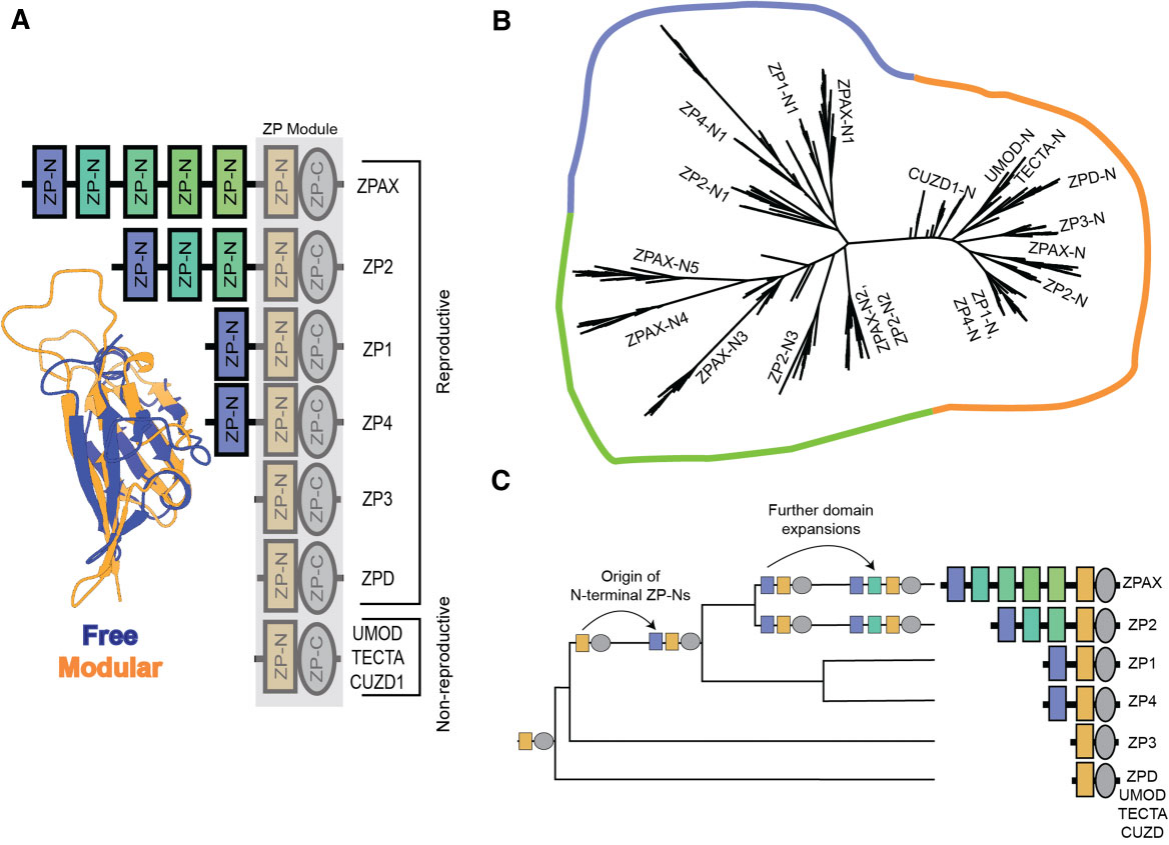
Phylogenetic analysis of ZP-N domain duplication history. (*A*) A structural alignment of mouse ZP2-N1 and ZP3-N highlights the broad structural conservation of these two classes of ZP-N domains (RMSD = ∼4.7 Å) despite only ∼18% amino acid sequence identity. The protein schematics summarize the ZP proteins included in this analysis. (*B*) Phylogenetic analysis (Kozlov et al. 2019) of ZP-N sequences (shown as a maximum likelihood tree) supports an ancestral separation between free and modular ZP-N domains (∼78% support). (*C*) A summary of ZP-N domain evolution based on the gene tree in (*B*). The ancestral protein contained a ZP module with a C-terminal ZP-N and ZP-C domains, and duplication of the ZP-N produced the most N-terminal domain found in ZP1, ZP4, ZP2, and ZPAX. Later duplication events within ZP2 and ZPAX gave rise to multiple additional ZP-N domains between ZP-N1 and the ZP module.

Although both ZP-N and ZP-C are immunoglobulin-like domains with a core β-sandwich (Bokhove and Jovine 2018), they are evolutionarily distinct domains that have low amino acid sequence identity, unique disulfide patterns, and variable loop structures (Lin et al. 2011). Independent ZP-C domains outside of the ZP module have been identified in *Caenorhabditis elegans* (Weadick 2020), and four of the egg coat proteins (ZP1, ZP2, ZP4, and ZPAX) contain additional ZP-N domains independent of the ZP-N/ZP-C pair in the ZP module (fig. 1*A*). We do not know of nonreproductive proteins that contain duplicated ZP-N domains. We refer to ZP-N domains in the ZP module as “modular” and the N-terminal repeats as “free” domains. As ZP-N domains can form asymmetric dimers through their β-sandwich edges (Jovine et al. 2002; Bokhove and Jovine 2018; Litscher and Wassarman 2020), they have been considered the major driver of ZP module polymerization. Although free ZP-N domains may similarly function as polymerization units, recent structural studies support that they may have acquired novel functions: the free ZP-N domains of ZP1 form intermolecular cross-links important for an egg coat structure (Nishimura et al. 2019), whereas N-terminal domains in ZP2 (Avella et al. 2013, 2014) and ZP4 (Dilimulati et al. 2022) have been implicated in sperm–egg binding. The functional diversification of duplicated ZP-N domains seems to play an important role in the evolution of species-specific interactions. Despite their functional significance, the evolutionary history of ZP-N domains within and between these many paralogous proteins has not been examined. Our combination of phylogenetic and machine learning approaches addresses how a complex history of whole gene and tandem domain duplications followed by structural adaptation produced the current diversity of ZP proteins.

## Results and Discussion

We investigated the evolutionary history of vertebrate ZP-N domains by extracting a total of 2,405 ZP-N domain sequences from ZP module–containing genes of 247 species with both reproductive (*zp1*, *zp2*, *zp3*, *zp4*, *zpax*, and *zpd*) and nonreproductive (*umod*, *tecta*, *cuzd1*) functions (supplementary table S1, Supplementary Material online). Although modular and free ZP-N sequences were found to share little sequence identity beyond four conserved cysteine residues that form stabilizing disulfide bonds, both domain types were highly similar in a 3D structure (fig. 1*A*). As such, we used a structure-based sequence alignment (Pei et al. 2008) to perform phylogenetic analysis. Maximum likelihood–based phylogenies indicated that the free ZP-N domains formed a single clade distinct from the ZP-C-associated modular ZP-N domains (fig. 1*B*), and this separation was robust to amino acid substitution matrices (LG, WAG, and JTT) (supplementary fig. S1, Supplementary Material online). The topology of the modular ZP-N clade was broadly consistent with previously published gene trees based on the complete ZP module with both ZP-N and ZP-C (Claw and Swanson 2012; Feng et al. 2018). The topology of the free ZP-N clade supported that the initial duplication gave rise to the first repeat of the tandem array shared by ZP1, ZP2, ZP4, and ZPAX, which was followed by lineage-specific repeat expansions of free ZP-Ns in *ZP2* and *ZPAX* (fig. 1*C*). Free ZP-Ns have only been identified in proteins associated with the egg coat.

**Fig. 2.**
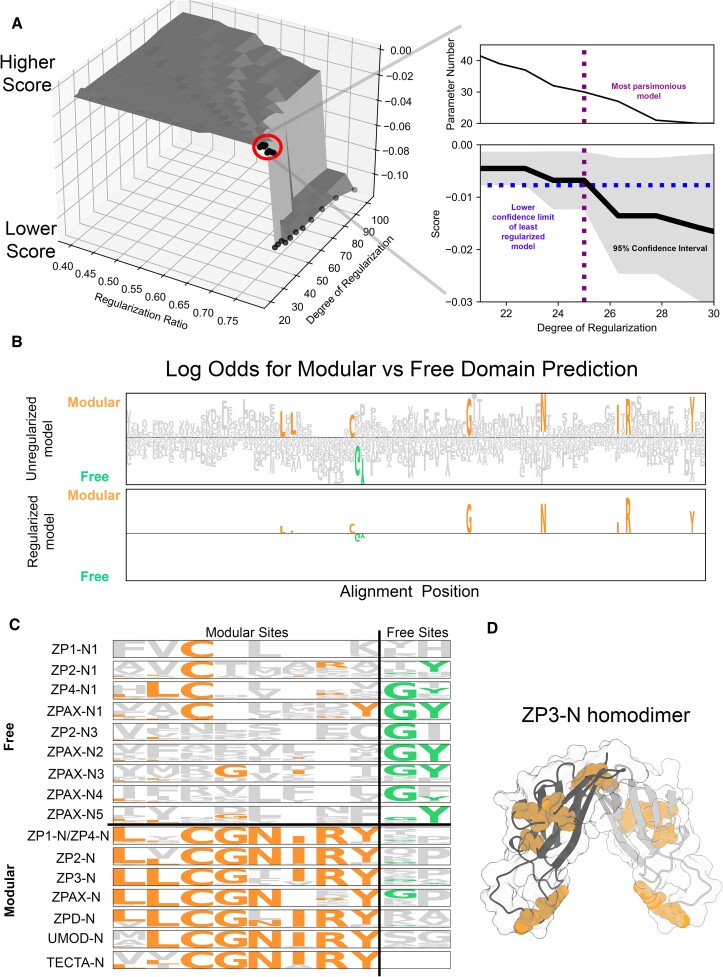
Machine learning–based inference of sequence features that distinguish modular and free ZP-N domains. A logistic regression model with elastic net regularization was trained on the ZP-N multiple sequence alignment generated as part of the phylogenetic analysis, with the data partitioned for training and testing (75% and 25%, respectively), with five-way cross-validation of the training data employed to estimate the error distribution of the score function. We defined our optimal model as the most parsimonious model (i.e., the fewest parameters) within the estimated 95% confidence interval of the unregularized model. (*A*) The space of regularization hyperparameters was explored during model optimization, plotted as a 3D surface (left). The score is the negative mean-squared error, and the dots correspond to the 2D cross-section shown on the right, with the blue line denoting the intersection between the lower confidence limit of the unregularized model to its intersection with the score as a function of regularization strength. (*B*) Comparison of the unregularized and optimal logistic regression models as LOGO plots with the height of each amino acid at each position corresponding to its parameter weight, with colored amino acids denoting parameters retained in the regularized model (orange for modular; green for free). Each parameter weight approximating the logs odd ratio for a modular domain prediction, when a residue is present at that position. (*C*) Sequence LOGOs were constructed for individual clades within the phylogeny. They emphasize the conservation of residues within the modular ZP-N clade. There is also greater conservation of a characteristic ZP-N disulfide bond in the most N-terminal ZP-Ns compared with other free domains. (*D*) Mapping highly predictive sites onto ZP-N protein models suggest differences in structural properties between free and modular domains. The available crystal structure ZP3-N (3d4c) was used and modeled as a dimer for spatial context. Modular-associated sites are generally buried along the outer edge of the homodimer.

The phylogenetic separation of modular and free ZP-N domains using a structure-based alignment suggests important structural differences between the two domain types, but their high sequence divergence has complicated a manual identification of such characteristics. Machine learning methods have been applied to various aspects of protein biology such as function prediction (Yang et al. 2018; Bonetta and Valentino 2020) and the classification of membrane-bound proteins (Guo et al. 2019). Here, we used a machine learning–based classification strategy to identify what structural features distinguish free and modular types of ZP-N domains. We applied a logistic regression model to the structurally aligned ZP-N domain sequences, where the probability of being a modular versus free ZP-N type was estimated for each of the 20 amino acids at each position in the alignment. Given the large number of parameters in this model (9,321), we combined elastic net regularization and cross-validation to identify the most parsimonious model (i.e., the fewest nonzero parameters) within the 95% confidence interval of the highest-scoring model (fig. 2*A and B*). Through this regularization strategy, we identified eight modular-associated and two free-associated residues that were sufficient to predict whether a given ZP-N sequence was modular or free with 100% accuracy (fig. 2*B*). The greater number of modular-associated residues and their greater probabilistic weight suggest a greater sequence conservation of modular domains (fig. 2*B*). A further examination of individual clades of modular and free ZP-Ns demonstrates the substantial sequence conservation of our residues identified by machine learning (fig. 2*C*).

An examination of the residues associated with either ZP-N type in the context of 3D structures suggests differences in both function and quaternary structural dynamics. ZP-N monomers have an immunoglobulin-like β-sandwich fold with the 4- and 3-membered β-strands connected by a disulfide bridge on each edge of the molecule. Biochemical and crystallographic studies support that modular ZP-N domains form asymmetric dimers through the molecular edge that includes the most N- and C-terminal β-strands (Jovine et al. 2006; Bokhove et al. 2016). Free ZP-N domains do not appear to dimerize through this N/C-terminal edge and have experienced functional diversification of the outer edge of the molecule to perform additional protein binding functions (Raj et al. 2017; Nishimura et al. 2019). When the modular-associated sites were mapped onto their respective structures, we observed that modular-associated residues formed an integrated network of mostly hydrophobic stabilizing contacts that interlocked between the β-sheets around the outer edge of the molecules (fig. 2*D*, supplementary fig. S2, Supplementary Material online). The phylogenetic clustering of free ZP-N domains (fig. 1*C*), along with molecular dynamics, supports the loss of dimerization activity along the free ZP-N lineage, which could have facilitated their evolution of new binding partners (supplementary fig. S3, Supplementary Material online). The stabilizing contacts along the outer edge of the modular ZP-N domains are consistent with these domains principally having structural roles, whereas in free domains, this edge has diversified to allow functional innovation. A further subdivision of free ZP-N domains by their major clades (the first repeat vs. internal repeats in ZP2 and ZPAX) largely supports our initial findings (supplementary fig. S4, Supplementary Material online). Consequently, our sequence-based machine learning classifier identified conserved residues underlying structural differences between the two domain types that have implications on their respective functions.

**Fig. 3.**
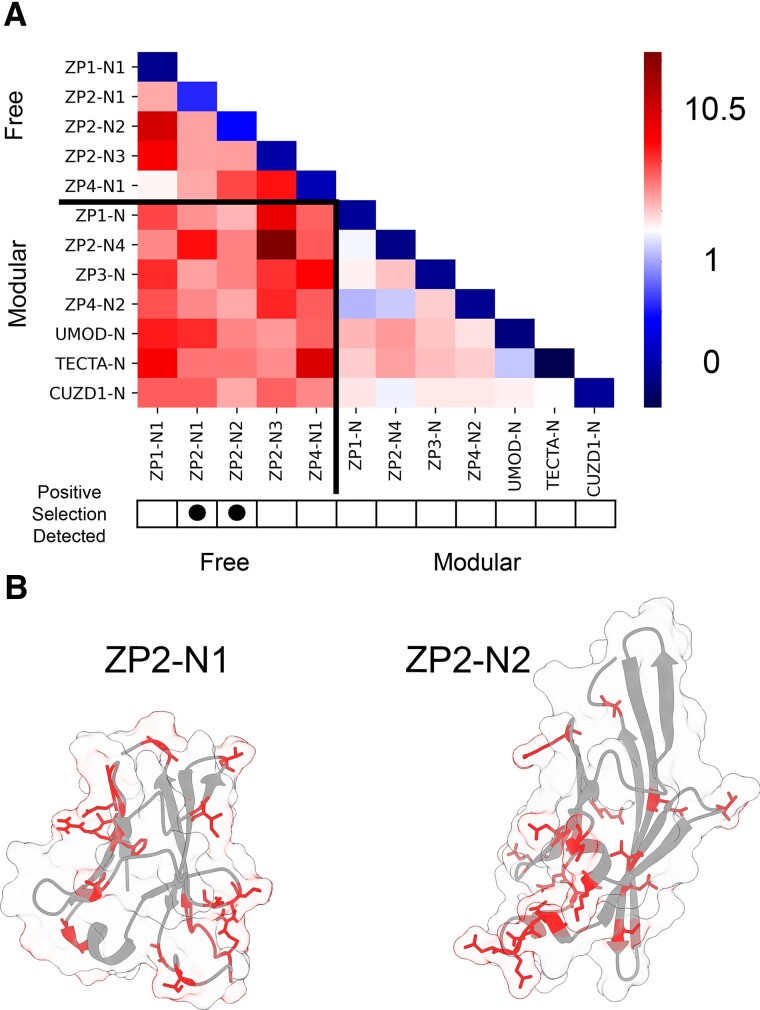
Amino acid diversity and tests of positive selection in modular and free ZP-N domains. (*A*) A heatmap showing the within-group and between-group mean phylogenetic distances for the orthologous groups of ZP-N domains (Kumar et al. 2018). (*B*) Positively selected sites in mammalian ZP2-N1 and ZP2-N2 were identified through maximum likelihood analysis and mapped onto protein models (4wrn for ZP2-N1 and an AlphaFold prediction for ZP2-N2) (Yang 2007).

The difference in the relative conservation of modular domain structures motivated an additional analysis of the sequence evolution of these ZP-N domains. Here, we focused on mammalian ZP genes (*zp1*, *zp2*, *zp3*, *zp4*, *umod*, *tecta*, and *cuzd1*) due to both higher genomic assembly quality and to avoid synonymous substitution saturation that may occur when considering greater phylogenetic breadth (Anisimova and Liberles 2012). Measures of sequence diversity within and between ZP-N groups reveal that modular domains are less diverse overall, and that free ZP-Ns are just as dissimilar to one another as they are to modular domains (fig. 3A).

These findings motivated molecular evolutionary analyses on 12 mammalian ZP-N domains, and only ZP2-N1 and ZP2-N2 showed evidence of positive selection (supplementary table S2, Supplementary Material online). These were notably the two domains with the lowest within-group similarity (diagonal of fig. 3*A*). Positively selected sites in ZP2-N1 were far from the homodimerization edge and physically closer to the network of modular-biased residues (fig. 3B). Analyses that detect positive selection in free ZP-Ns may reveal complementary information to the high conservation of residues in modular ZP-Ns. Protein regions associated with structural stability in modular domains may rapidly evolve in free domains that gain a new binding interface. Positively selected sites also constituted a substantial portion of the solvent exposed surface area (34% in ZP2-N1 and 24% in ZP2-N2), potentially facilitating their evolution of novel functions and protein interactions. The rapid evolution of ZP2-N1 is consistent with its role in species-specific sperm recognition (Avella et al. 2014) and may reflect sexual coevolution with its sperm receptor (whose identity is currently unknown). Remarkably, these positively selected sites cluster near a region associated with species-specific sperm protein binding in free invertebrate ZP-N domains (Raj et al. 2017). However, based on expansion and retraction of loop lengths outside the core β-sandwich, we believe that these invertebrate free ZP-N domains evolved independently of the free ZP-N domains of vertebrates, suggesting that the expansion of ZP-N arrays for species-specific sperm recognition is a convergent phenomenon that has arisen multiple times throughout metazoan evolution. Previously, positive selection in ZP3 had been detected in certain mammalian clades (Swanson et al. 2001; Turner and Hoekstra 2006). However, selective pressures related to reproduction (e.g., mating system) can vary across taxa, and this can affect why a broad search of Boreoeutherian mammals did not detect positive selection in ZP3 when compared with previous analyses on limited taxon data sets. Similar observations have been made in the fertilization proteins Izumo and Juno (Grayson 2015).

In summary, our combined phylogenetic, machine learning classification and positive selection analyses illustrated a clear distinction between modular and free ZP-N domains. These two classes of domains experienced different evolutionary trajectories, as modular ZP-Ns likely retained a conserved structural role, whereas free ZP-Ns neofunctionalized to serve different reproductive functions. These findings are of relevance to the evolution of species specificity in fertilization, as the ZP-N domain expansion of ZP2 provided substrates to evolve novel species-specific interactions. Structural changes within free ZP-Ns could result in a dimerization edge and the evolution of a new sperm binding loop. As these domains are coopted into a reproductive context, coevolution (Clark et al. 2009; Hart et al. 2018) and sexual conflict (Gavrilets and Waxman 2002) with sperm proteins could contribute to their rapid evolution. This reflects the evolutionary dynamics that drive the structural diversification and neofunctionalization of duplicated domains. Our combined phylogenetic and machine learning approach outlined here can be applied to other essential gene families with complex duplication histories.

## Materials and Methods

### Multiple Sequence Alignment

Sequences for multiple ZP-N containing proteins were curated from the Ensembl database (release 104) (Howe et al. 2021). Sequences were preliminarily labeled as one of the ZP genes of interest based on PSI-BLAST *e*-value scores (Altschul et al. 1997). Sets of orthologous genes were aligned with multiple alignment using fast fourier transform (MAFFT) (Katoh and Standley 2013) and then trimmed to individual ZP-N domains. Groups of orthologous ZP-N domains were deemed “orthogroups.” Sequences with ambiguous characters were removed, and then sets of orthologous ZP-N sequences were realigned with MAFFT. A full multiple sequence alignment was generated by concatenating orthogroup alignments together using a representative paralog alignment: individual representative sequences were selected from each orthogroup and aligned using the structural-based PROMALS tool (Pei et al. 2008). This approach was used because of the low sequence identity but the high structural similarity between paralogous Z-N domains. A custom script was used to algorithmically add gaps to orthogroup alignments to form a full multiple sequence alignment. For phylogenetics, CD-Hit was used to remove highly clustered and highly similar sequences (>90% identity) (Li and Godzik 2006; Fu et al. 2012), in order to improve computing speed, and also because this study was not concerned with very recent evolutionary splits. A full data set was used for machine learning training because such methods are less computationally strained by large alignments and can gain greater sensitivity with a high depth of taxonomic sampling.

### Phylogenetics

Maximum likelihood phylogenies were built using RAxML-NG (Kozlov et al. 2019), and multiple different amino acid substitution matrices were tested (LG + G, JTT + G, WAG + G), to evaluate the robustness of the deepest phylogenetic divide. The maximum likelihood tree was selected from 100 replicate runs using different starting trees. Nodal support was calculated with transfer bootstrap expectation (Lemoine et al. 2018), a modified form of bootstrapping that is more effective at detecting deep phylogenetic relationships in data sets with a large number of taxa. Sequence labels were initially based on BLAST results but later refined based on phylogenetic clustering (e.g., ZP1-N1, ZP2-N1, ZP4-N1).

### Machine Learning

A basic machine learning algorithm using mean-squared regression and regularization was coded in Python to distinguish the two free and modular groups of ZP-N domains. Logistic regression models are well suited for these classifications, because their outputs are bounded between 0 and 1, which can be interpreted as a probability that a given domain is modular (Bewick et al. 2005). The multiple sequence alignment was identical to that used for phylogenetic analysis. The alignment was split into a testing (25%) and training set (75%), and logistic regression modeling with cross-validation was performed on the training set using five-way cross-validation. The final model scores were based on performance in the testing data set.

For machine learning analysis, aligned ZP-N sequences were one-hot encoded: each position in the sequence was converted into a vector of 20 digits, corresponding to the 20 amino acids. The value was set to 1 for the entry in the vector corresponding to that residue, and all other values were set to 0. Gapped sites were set to a vector of 20 0’s. Thus, the classifier was trained using (1 + 20*n*) features (there is an additional intercept term), where *n* is the alignment length. Each of these features had a parameter associated with it, and the value of the parameter indicated how informative that feature was, and whether it supported a modular ZP-N or free ZP-N classification. There were a large number of possible parameters in this model (9,321 including the intercept), which introduced a risk for “overfitting” (Hawkins 2004) and, thus, motivated our regularization strategies.

To determine the minimal number of highly informative parameters, elastic net regularization was employed to penalize overparameterization and reduce overfitting (Zou and Hastie 2005). In our sci-kit learn implementation (Pedregosa et al. 2011), both the strength of regularization and the L1/L2 penalty ratio between the two penalty types were optimized by grid search. The highest-scoring model was identified according to the negative mean-squared error scoring metric. In order to choose a suitable sparse model (i.e., fewest nonzero parameters), we adapted the one standard error rule common in machine learning (Hastie et al. 2009), where the sparsest model that is still within one standard error of the highest-scoring model is selected. For this analysis, we used 95% confidence intervals (∼1.96 standard errors) to identify the sparsest model (fewest nonzero parameters) that was not statistically different from the highest-scoring model sampled. Raw parameter values were plotted in the style of sequence LOGO plots (Schneider and Stephens 1990). The sum of the raw parameter values for matching amino acids in the alignment (and the intercept term) was equivalent to the log odds that a given sequence was classified as modular. For the sake of simplicity, each parameter was described as the log odds associated with a particular residue. In addition to the initial binary classification (free vs. modular), our analysis was repeated using a three-way multiclassification (first N-terminal, internal, and modular). This procedure used alignments, hyperparameter grid searching, and regularization strategies in the same manner as the binary classification.

### Sequence Divergence and Positive Selection Analyses

Our analyses of sequence divergence and positive selection were performed on a set of Boreoeutherian mammals, and we used the mammalian ZP-N domains coming from *zp1*, *zp2*, *zp3*, *zp4*, *umod*, *tecta*, and *cuzd1*. Boreoeutherian sequences were mined from Ensembl (Howe et al. 2021) and were included in these analyses if they were present in 10 or more of these ZP-N domain orthogroups. Phylogenetic distances both within and between the orthogroups were calculated in MEGA using Poisson estimation with a gamma distribution of variation between sites (Kumar et al. 2016, 2018).

Evidence of positive selection was measured using PAML analyses (Yang et al. 2005, 2007) on the same sets of ZP-N domains from the sequence divergence estimation. A likelihood ratio test between a model allowing positive selection (M8) and a neutral model (M8a) was used to determine which domains showed evidence of positive selection. Likelihood ratio tests were performed by comparing M8 and M8a, using a χ^2^ distribution with one degree of freedom. We also performed a Benjamini–Hochberg *P*-value correction to account for multiple testing (Benjamini and Hochberg 1995). Positively selected sites were visualized on a published crystal structure (ZP2-N1) (Raj et al. 2017) or the alpha-fold predicted structure (Jumper et al. 2021) when this did not exist (ZP2-N2). Sites were labeled if they had a posterior probability of being positively selected >75% according to Bayes Empirical Bayesian analysis.

### Visualization and Other Methods

When protein structures were not available, Alpha-Fold2 tertiary structure prediction was used (Jumper et al. 2021), and 3D protein structures were visualized using either pymol (Schrödinger 2015) or ChimeraX (Pettersen et al. 2004). Docking simulations of homodimerization for ZP2-N1 and ZP3-N were performed using Rosetta 3.5 (Chaudhury and Gray 2008; Sircar et al. 2010). Briefly, each template structure was energy-minimized in Rosetta using the relax function, each structure was duplicated, aligned to the dimeric ZP-N structure of UMOD (PDB 4wrn), 10,000 independent docking simulations were performed, and interface scores were analyzed for the top 5% lowest energy structures.

## Supplementary Material

msac105_Supplementary_Data

## Data Availability

We are sharing a link to a github repository that contains our maximum likelihood phylogeny and relevant alignments and code. The repository link is https://github.com/amrivera526/ZPN_Evolution.
