# Supplementary material for: Domain Expansion and Functional Diversification in Vertebrate Reproductive Proteins
Source: Mol Biol Evol. 2022 May 19;39(5):msac105. doi: 10.1093/molbev/msac105 (PMC9154058; doi:10.1093/molbev/msac105)
Supplement: msac105_Supplementary_Data [file msac105_supplementary_data.pdf]

Materials and Methods

Multiple Sequence Alignment

Sequences for multiple ZP-N containing proteins were curated from the Ensembl database (release 104) (Howe et al. 2021). Sequences were preliminarily labelled as one of the ZP genes of interest based on PSI-BLAST e-value scores (Altschul et al. 1997). Sets of orthologous genes were aligned with MAFFT (Katoh and Standley 2013) and then trimmed to individual ZP-N domains. Groups of orthologous ZP-N domains were deemed “orthogroups”. Sequences with ambiguous characters were removed, and then sets of orthologous ZP-N sequences were realigned with MAFFT. A full multiple sequence alignment was generated by concatenating orthogroup alignments together using a representative paralog alignment:individual representative sequences were selected from each orthgroup, and aligned using the structural based PROMALS tool (Pei et al. 2008). This approach was used because of the low sequence identity, but high structural similarity between paralogous Z-N domains. A custom script was used to algorithmically add gaps to orthogroup alignments to form a full multiple sequence alignment. For phylogenetics, CD-Hit was used to remove highly cluster highly similar sequences (>90% identity) (Li and Godzik 2006; Fu et al. 2012), in order to improve computing speed, and because this study was not concerned with very recent evolutionary splits. A full dataset was used for machine learning training because those methods are less computationally strained by large alignments and can gain greater sensitivity with a high depth of taxonomic sampling.

Phylogenetics

Maximum likelihood phylogenies were built using RAxML-NG(Kozlov et al. 2019), and multiple different amino acid substitution matrices were tested (LG+G, JTT+G,WAG+G), to evaluate the robustness of the deepest phylogenetic divide. The maximum likelihood tree was selected from 100 replicate runs using different starting trees. Nodal support was calculated with transfer bootstrap expectation (Lemoine et al. 2018), a modified form of bootstrapping that is more effective at detecting deep phylogenetic relationships in datasets with large number of taxa. Sequence labels were initially based on BLAST results but later refined based on phylogenetic clustering, (e.g. ZP1-N1, ZP2-N1, ZP4-N1).

Machine Learning

A basic machine learning algorithm using mean squared regression and regularization was coded in Python to distinguish the two free and modular groups of ZP-N domains. Logistic regression models are well suited for these classifications, because their outputs are bounded between 0 and 1, which can be interpreted as probability that a given domain is modular (Bewick et al. 2005). The multiple sequence alignment was identical to that used for phylogenetic analysis. The alignment was split into a testing (25%) and training set (75%), and logistic regression modelling with cross-validation was performed on the training set using five-way cross validation. The final model scores were based on performance in the testing dataset.

For machine learning analysis, aligned ZP-N sequences were one-hot encoded: each position in the sequence was converted into a vector of twenty digits, corresponding to the twenty amino acids. The value was set to 1 for the entry in the vector corresponding to that residue, and all other values are set to 0. Gapped sites were set to a vector of twenty 0’s. Thus, the classifier was trained using (1+20n) features (there is an additional intercept term), where n

is the alignment length. Each of these features has a parameter associated with it and the value of the parameter indicates how informative that feature is, and whether it supports a modular ZP-N or free ZP-N classification. There are a large number of possible parameters in this model (9321 including the intercept), ,which introduces a risk for "overfitting"(Hawkins 2004), and motivates our regularization strategies.

To determine the minimal number of highly informative parameters, elastic net regularization was employed to penalize overparameterization and reduce overfitting (Zou and Hastie 2005). In our sci-kit learn implementation (Pedregosa et al. 2011), both the strength of regularization and the L1/L2 penalty ratio between the two penalty types were optimized by grid search. The highest scoring model was identified according to the negative mean-squared error scoring metric. In order to choose a suitable sparse model (i.e. fewest non-zero parameters), we adapted the one standard error rule common in machine learning (Hastie et al. 2009), where the sparsest model that is still within one standard error of the highest scoring model is selected. For this analysis we used 95% confidence intervals (~1.96 standard errors), to identify the sparsest model (fewest non-zero parameters) that is not statistically different from the highest scoring model sampled. Raw parameter values were plotted in the style of sequence LOGO plots (Schneider and Stephens 1990). The sum of the raw parameter values for matching amino acids in the alignment (and the intercept term), are equivalent to the log odds that a given sequence is classified as modular. For simplicity, each parameter is described as the log odds associated with a particular residue. In addition to the initial binary classification (free vs modular), our analysis was repeated using a three-way multiclassification (first N-terminal, internal, and modular). This procedure used alignments, hyperparameter grid searching, and regularization strategies in the same manner as the binary classification.

Sequence Divergence and Positive Selection Analyses

Our analyses of sequence divergence and positive selection was performed on a set of Boreutherian mammals., and we used the mammalian ZP-N domains coming from *zp1*, *zp2*, *zp3*, *zp4*, *umod*, *tecta*, and *cuzd1*. Boreoeutherian sequences were mined from Ensembl (Howe et al. 2021), and were included in these analyses if they were present in 10 or more of these ZP-N domain orthogroups. Phylogenetic distances both within and between orthogroups were calculated in MEGA using Poisson estimation with a gamma distribution of variation between sites (Kumar et al. 2016; Kumar et al. 2018).

Evidence of positive selection was measured using PAML analyses (Yang et al. 2005; Yang 2007) on the same sets of ZP-N domains from the sequence divergence estimation. A likelihood ratio test between a model allowing positive selection (M8) and a neutral model (M8a), was used to determine which domains showed evidence of positive selection. Likelihood ratio tests were performed by comparing M8 and M8a, using a chi-squared distribution with one degree of freedom. We also performed a Benjamini-Hochberg p-value correction to account for multiple testing (Benjamini and Hochberg 1995). Positively selected sites were visualized on a published crystal structure (ZP2-N1) (Raj et al. 2017), or the alpha-fold predicted structure (Jumper et al. 2021 Jul 15) when this did not exist (ZP2-N2). Sites were labelled if they had a posterior probability of being positively selected > 75% according Bayes Empirical Bayesian (BEB) analysis.

Visualization and Other methods

When protein structures were not available Alpha-Fold2 tertiary structure prediction was used (Jumper et al. 2021), and three-dimensional protein structures were visualized using either pymol (Schrödinger 2015) or ChimeraX (Pettersen et al. 2004). Docking simulations of homodimerization for ZP2-N1 and ZP3-N were performed using Rosetta 3.5 (Chaudhury and Gray 2008; Sircar et al. 2010). Briefly, each template structure was energy minimized in Rosetta using the relax function, each structure was duplicated, aligned to the dimeric ZP-N structure of uromodulin (PDB 4wrn), 10000 independent docking simulations performed, and interface scores analyzed for the top 5% lowest energy structures.

Data Availability

We are sharing a link to a github repository that contains our maximum likelihood phylogeny and relevant alignments and code. The repository link is https://github.com/amrivera526/ZPN_Evolution

	Mammals	Birds	Reptiles	Amphibians	Fish
ZP1-N1	50 (30)	28 (11)	12 (10)	0 (1)	0 (19)
ZP1-N	53 (30)	28 (9)	11 (8)	0 (0)	28 (20)
ZP2-N1	70 (46)	35 (20)	8 (4)	2 (2)	36 (34)
ZP2-N2	74 (46)	44 (19)	14 (10)	1 (1)	19 (21)
ZP2-N3	75 (45)	41 (18)	15 (11)	2 (2)	3 (3)
ZP2-N	76 (38)	40 (7)	14 (10)	1 (1)	15 (14)
ZP3-N	72 (36)	40 (13)	19 (12)	2 (2)	53 (38)
ZP4-N1	63 (37)	33 (11)	12 (10)	2 (2)	34 (23)
ZP4-N	52 (29)	35 (8)	12 (8)	1 (1)	61 (42)
ZPAX-N1	2 (2)	22 (9)	12 (11)	2 (2)	67 (50)
ZPAX-N2	2 (2)	24 (9)	13 (9)	2 (2)	67 (53)
ZPAX-N3	2 (2)	25 (9)	12 (9)	2 (2)	64 (51)
ZPAX-N4	2 (2)	20 (8)	12 (7)	2 (2)	69 (56)
ZPAX-N5	2 (2)	24 (12)	15 (11)	2 (2)	68 (55)
ZPAX-N	2 (2)	13 (5)	8 (8)	2 (2)	59 (44)
ZPD-N	0 (0)	38 (9)	18 (10)	2 (2)	69 (54)
UMOD-N	75 (41)	0 (0)	6 (6)	1 (1)	8 (6)
TECTA-N	43 (1)	4 (1)	11 (0)	1 (0)	49 (2)
CUZD1-N	62 (38)	44 (23)	17 (13)	2 (2)	51 (37)

Summary of species sampled in phylogeny

Table S1: This table summarizes the sequences included for the machine learning classification and phylogenetic analysis (these values are in parenthesis). There were 2405 ZP-N sequences across 247 species included in the machine learning analysis and the phylogenetics included 1488 ZP-N sequences from 210 species. The phylogeny was filtered for sequences at greater than 90% identity, unlike the machine learning dataset and there are additional differences due to manual filtering of alignments. Here the amphibians only included frogs due to genomic availability reasons.The fish class includes all non-tetrapod vertebrates, and is non-monophyletic. All labels in this table are based on BLAST results Labels in the final phylogeny are based on phylogenetic clustering.

Domain	M8a	M8	-2ΔlogL	*p* value	*p* value corrected
CUZD1-N	p_0 = 0.97, p_1= 0.03, p = 0.64, q=2.1, ω = 1	p_0 = 0.99, p_1= 0.01, p = 0.63, q=1.9, ω = 2.5	2.4	0.06	0.25
TECTA-N	p_0 = 0.98, p_1= 0.02, p = 0.05, q=0.99, ω = 1	p_0 = 0.99, p_1= 0.01, p = 0.05, q=0.97, ω = 1.9	1.0	0.16	0.24
UMOD-N	p_0 = 0.98, p_1= 0.02, p = 0.29, q=0.61, ω = 1	p_0 = 0.98, p_1= 0.02, p = 0.29, q=0.61, ω = 1	0.0	0.50	0.57
ZP1-N1	p_0 = 0.74, p_1= 0.26, p = 0.74, q=1.4, ω = 1	p_0 = 0.99, p_1= 0.01, p = 0.54, q=0.51, ω = 3.5	1.9	0.08	0.19
ZP1-N2	p_0 = 1, p_1= 0, p = 0.46, q=0.68, ω = 1	p_0 = 1, p_1= 0, p = 0.46, q=0.68, ω = 1	0.0	0.50	0.57
ZP2-N1	p_0 = 0.43, p_1= 0.57, p = 2.7, q=8.0, ω = 1	p_0 = 0.65, p_1= 0.35, p = 1.2, q=1.4, ω = 1.5	6.7	4.7E-03	0.03 *
ZP2-N2	p_0 = 0.42, p_1= 0.58, p = 29.5, q=99, ω = 1	p_0 = 0.70, p_1= 0.30, p = 1.6, q=1.5, ω = 1.9	18.8	7.2E-06	8.6E-5 *
ZP2-N3	p_0 = 0.67, p_1= 0.33, p = 1.6, q=7.9, ω = 1	p_0 = 0.85, p_1= 0.15, p = 0.65, q=1.5, ω = 1.4	1.5	0.11	0.22
ZP2-N	p_0 = 0.87, p_1= 0.13, p = 0.76, q=3.4, ω = 1	p_0 = 0.87, p_1= 0.13, p = 0.76, q=3.4, ω = 1	0.0	0.50	0.57
ZP3-N	p_0 = 0.98, p_1= 0.02, p = 0.55, q=1.2, ω = 1	p_0 = 1, p_1= 0, p = 0.52, q=1.21 ω = 2.3	0.0	0.50	0.57
ZP4-N1	p_0 = 0.58, p_1= 0.42, p = 1.3, q=4.3, ω = 1	p_0 = 0.93, p_1= 0.07, p = 0.64, q=0.70, ω = 1.9	2.3	0.065	0.19
ZP4-N	p_0 = 0.82, p_1= 0.18, p = 0.40, q=1.1, ω = 1	p_0 = 0.94, p_1= 0.06, p = 0.37, q=0.67, ω = 1.5	1.1	0.15	0.26

Summary of PAML Output

Table S2: This table summarizes the results from PAML analysis (Yang 2007). Here we compared a neutral model (M8a) to a model that allows positive selection (M8). A * denotes statistically significant p values after Benjamini-Hochberg multiple testing correction (Benjamini and Hochberg 1995).

\

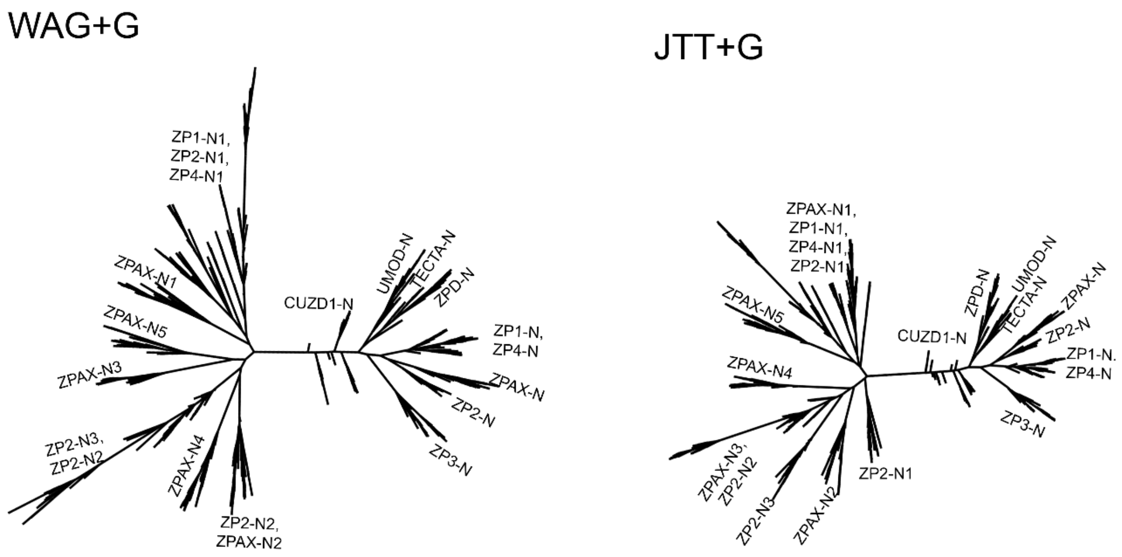

Phylogenies from alternative substitution matrices

Supplemental Figure 1: These two trees were produced through RAxML-NG(Kozlov et al. 2019). Major aspects of the tree are conserved, specifically the monophyletic grouping of free ZP-N domains.

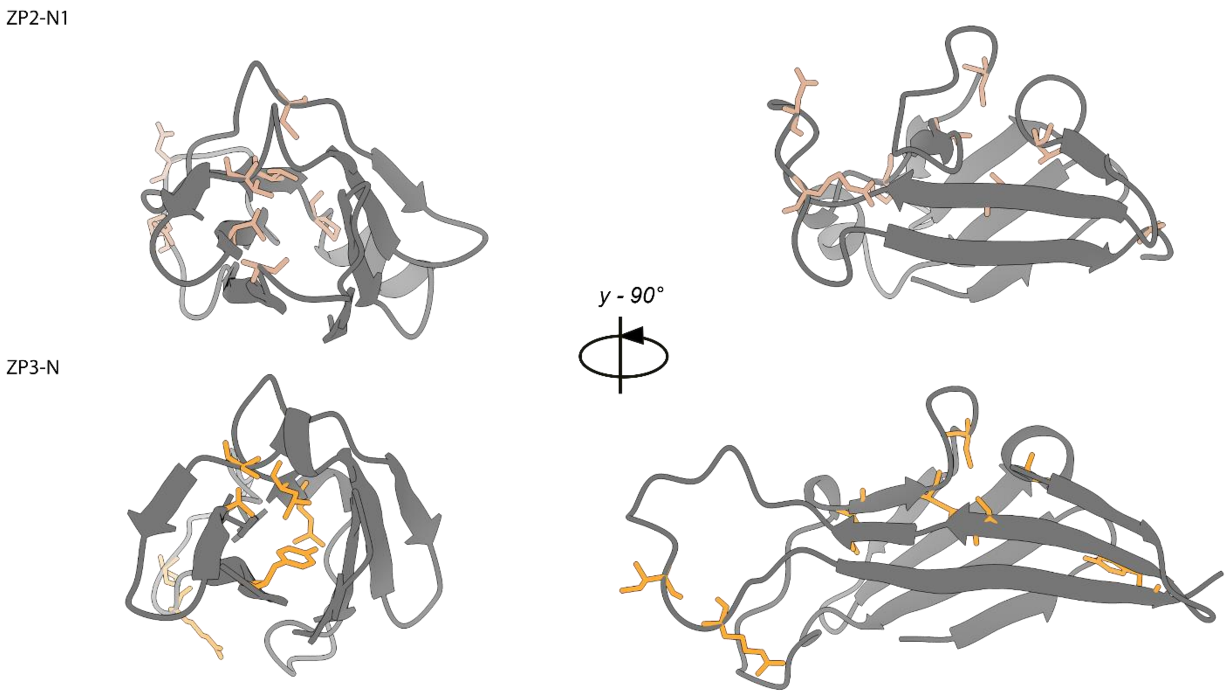

Modular-biased sites in the context of the ZP-N domain core

Supplemental Figure 2: This figure shows the modular biased sites in the context of both ZP2-N1 (Raj et al. 2017) and ZP3-N (Monne et al. 2008) crystal structures. The brighter orange corresponds to the modular-biased sites according to our machine learning model, while the duller orange on ZP2-N1 represent the structural homolog of those sites according to a pymol (Schrödinger 2015) structural alignment. We observe a clustering of these sites within the core of the domain in ZP3-N and not ZP2-N1. The two positions of each are 90° rotations around the y-axis.

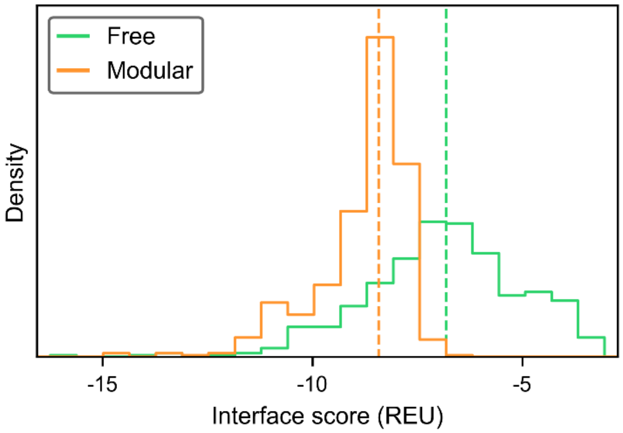

Rosetta docking simulation of dimerization

Supplemental Figure 3: This is a histogram of interface scores from rosetta docking simulations of dimers for both free (ZP2-N1) and modular (ZP3-N) ZP-N domains.

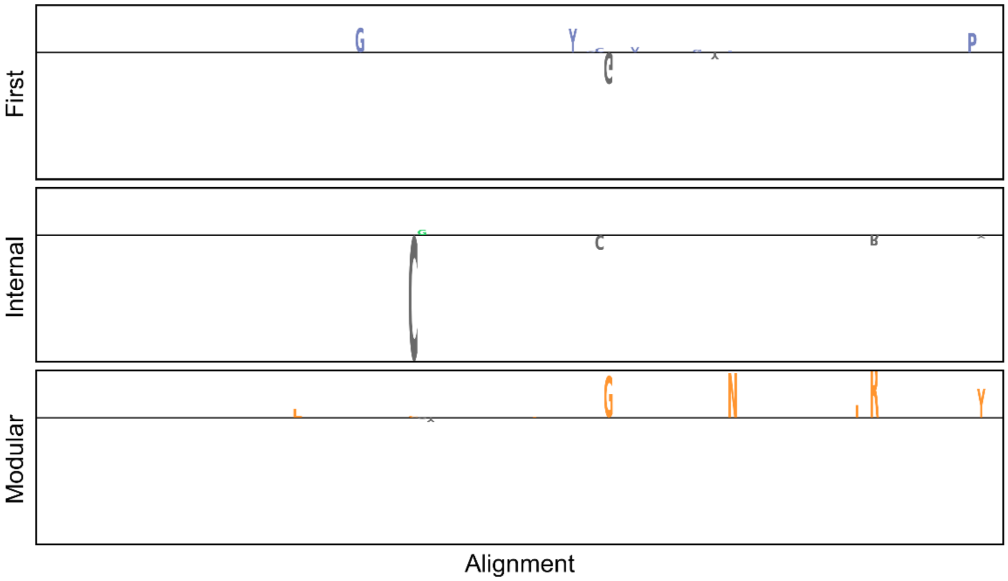

Multiclass machine-learning ZP-N domain classification

Supplemental Figure 4: Since we observed monophyletic grouping of the most N-terminal ZP-N domain, we performed a multiclass variation of our machine learning analysis. The data was split into three classes: modular, first (i.e. most N-terminal free domain), and internal domains (all other free ZP-N domains). In this multiclass analysis the model is fit three times, each time producing a classifier that distinguishes one of the classes from the rest of the data. The first row is the unregularized model from our two-class analysis, and our regularized multiclass models are summarized in the other three rows, where positive values suggest a bias towards that class. Our modular classifier mostly recapitulates our earlier results, because it is in essence still comparing modular ZP-Ns versus all free ZP-Ns. The first ZP-N domain recapitulates the two free associated residues andahas a few amino acids associated with it with relatively low parameter values. The internal ZP-N domains seem to have a bias against the cys 2-3 bond, but that likely is a reflection of the conservation of the second cysteine in the first ZP-Ns and the third cysteine in modular domains
